# Heterogeneity in Behaviour and Movement can Influence the Stability of Predator–Prey Periodic Travelling Waves

**DOI:** 10.1007/s11538-022-01101-8

**Published:** 2022-11-23

**Authors:** Renato Andrade, Christina A. Cobbold

**Affiliations:** 1 School of Mathematics and Statistics, University of Glasgow, Glasgow, G12 8QQ UK; 2 Boyd Orr Centre for Population and Ecosystem Health, University of Glasgow, Glasgow, G12 8QW UK

**Keywords:** Periodic travelling waves, Predator–prey, Reaction–diffusion, Homogenisation, Heterogeneity

## Abstract

Cyclic predator–prey systems are often observed in nature. In a spatial setting, these can manifest as periodic traveling waves (PTW). Environmental change and direct human activity are known to, among other effects, increase the heterogeneity of the physical environment, which prey and predator inhabit. Aiming to understand the effects of heterogeneity on predator–prey PTWs, we consider a one-dimensional infinite landscape Rosenzweig–MacArthur reaction–diffusion model, with alternating patch types, and study the PTWs in this system. Applying the method of homogenisation, we show how heterogeneity can affect the stability of PTW solutions. We illustrate how the effects of heterogeneity can be understood and interpreted using Turchin’s concept of residence index (encapsuling diffusion rate and patch preference). In particular, our results show that prey heterogeneity acts to modulate the effects of predator heterogeneity, by this we mean that as prey increasingly spend more time in one patch type over another the stability of the PTWs becomes more sensitive to heterogeneity in predator movement and behaviour.

## Introduction

Population cycles are one of the most studied aspects of populations dynamics (Barraquand et al. 2017), and such cycles can occur uniformly along the space that the species inhabit, or, alternatively, more complex spatiotemporal phenomena can be observed. One example of the latter happens in the red grouse population in northeast Scotland where population fluctuations in each location exhibited a decrease in synchronicity the further apart these locations were (Moss et al. (2000)). Another particular example studied by Lambin et al. (1998) and Berthier et al. (2014) for field voles in Kielder Forest in northern England, and in France, is a type of travelling wave observed in cyclic populations. In this case, populations in different spatial locations oscillate with the same temporal period, but exhibit a phase shift in space, giving the appearance of a wave with temporal oscillations in its wake. Such patterns are called periodic travelling waves (PTWs). PTWs happen when the populations move in a way that present periodicity both in space and time, with peaks and troughs of individuals moving across space at constant speed. A useful analogy is the wave-like phenomenon that spectators perform in a stadium during a crowded sport event, where they raise and lower their arms at similar moments to their neighbours in such a way that the net effect is a wave of raising and falling arms propagating around the stadium. Tenow et al. (2013) describes spatiotemporal data consistent with PTW for winter moth (*Operophtera brumata*) distributions in continental Europe. The study was able to estimate wavelength, speed and direction of the PTW, which exhibited decade-long periodic outbreaks. Sherratt and Smith (2008) review other past studies reporting populations possibly exhibiting PTW behaviour.

Spatiotemporal data is often challenging to obtain (Bennett and Sherratt 2017), since it involves a laborious and costly monitoring at several locations through usually extensive periods of time (Berthier et al. 2014). Hence, a motivation behind understanding the properties of PTWs comes from the possibility of improving our ability to track and predict the distribution of populations across space, e.g. in conservation field studies. Pest management can also benefit from an improved ability to track and control future outbreaks by increasing the efficiency of costly data collection (Petrovskii et al. 2014).

There are several frameworks for studying population dynamics in space, among which we highlight reaction–diffusion equations (Cantrell and Cosner 2004). They have been playing a fundamental role in modelling population movement since the work of Skellam (1951). Populations exhibiting PTWs can then be studied as solutions of reaction–diffusion models. Such solutions can be classified as either stable, with a regular spatiotemporal structure as described above, and unstable, which can manifest though irregular oscillations with no distinguishable shape (spatiotemporal chaos (Sherratt et al. 1995)). Populations displaying irregular spatiotemporal oscillations can be difficult to monitor and detect (Lambin et al. 1998). Therefore, by studying such reaction–diffusion models it is possible to better understand the underlying causes of spatiotemporal oscillations of natural populations. The seminal work of Kopell and Howard (1973) first determined the conditions for existence and stability of PTW solutions of coupled reaction–diffusion equations.

Amid the myriad of factors that may affect PTW stability, we investigate the effect of heterogeneity in the environment. In line with this thought, Johnson et al. (2004) argued how a mosaic-like heterogeneous environment alone can induce travelling waves in larch budmoth. There are many ways in which environmental heterogeneity affects populations. Different habitat types can be more or less suitable for population growth, and boundaries between habitat types can affect behaviour and movement (Schultz and Crone 2001; Bélisle and Desrochers 2002). For example, the grey shrike-thrush (*Colluricincla harmonica*) and the white-throated treecreeper (*Cormobates leucophaeus*) were found to act differently in forests when compared to how they act in open areas, even if they are adjacent, trying to avoid the latter moving through them more quickly (Robertson and Radford 2009). The predation risk of forest birds’ nests by a nest predator (red squirrel, *Tamiasciurus hudsonicus*) can also vary spatially depending on habitat quality (from the point of view of the predator) (Martin and Joron 2003).

Nonetheless, there have been a small number of studies examining the effect of environmental heterogeneity specifically on PTWs. We are then motivated by the question left open by Sherratt and Smith (2008): “What is the effect of spatial heterogeneity on periodic travelling waves?” Sieber et al. (2010) showed that small temporal noise in reaction–diffusion equations is able to supress PTWs. In contrast, we are interested in considering spatial heterogeneity. Connected with such interest is the work of Sherratt et al. (2003), who studied heterogeneity generated by obstacles in the landscape which could not be traversed by the species. They found that the shape and size of obstacles affect the wavelength of PTWs in a two-dimensional reaction–diffusion predator–prey system.

However, spatial heterogeneity can be observed in different ways, such as in habitat patches, i.e. regions with relative homogeneity but significantly different from their immediate surroundings, being more or less suitable for each of the species inhabiting them. For example, Gauduchon et al. (2013) studied the repercussions of heterogeneity by comparing the dynamics of two predator–prey models in a habitat composed of three one-dimensional patches. The three patches differed by the species’ parameters values from patch to patch. Their results showed that fragmentation via habitat loss can decrease cycle amplitude and average density of prey and predator populations. Shigesada et al. (1986) demonstrated that patch environmental heterogeneity in a single-species reaction–diffusion system could generate PTW solutions. Their analysis resulted in conditions for the population to either be able to invade the environment through a PTW or become extinct. Alternatively, Kay and Sherratt (2000) showed that environmental heterogeneity (through random spatial variation on the kinetic parameters) in a predator–prey system can allow persistence of PTWs that would otherwise die out in a finite domain. However, they did not consider the spatial heterogeneity in movement or the effects of habitat boundaries.

Many of the studies that consider heterogeneity via a collection of patches contemplate what we refer to as coarser grain environmental heterogeneity. By that we mean that patch size was not necessarily assumed to be small compared to the scale of movement rates. However, species can often encounter many habitat types during their lifetime and rapidly move through a landscape. Such a scenario can be thought of as finer grain heterogeneity, in which the effects of environmental heterogeneity can be studied through a landscape composed of a large number of small patches of different habitat types. One way to theoretically deal with such multiscale heterogeneity is via asymptotic homogenisation (Holmes 2012).

Our work is based on the previous studies of Yurk and Cobbold (2018) and Cobbold et al. (2022), who developed an asymptotic homogenisation framework for reaction–diffusion equations on a landscape composed of an infinite sequence of one-dimensional patches of two types. The patches differed in population dynamics, movement and the organism’s response to patch boundaries. They studied how environmental heterogeneities on the patch level (e.g. in the scale of meters) have an impact on landscape level (e.g. in the scale of kilometres) population densities. The focus species are assumed to move through the environment in a way that each individual passes through a substantial number of different patches during its lifespan, behaving differently in each patch type. This framework essentially corresponds to finding a systematic way of “averaging” the patch level heterogeneity, resulting in an approximate but potentially much simpler system of equations that describes the aggregate effect of the many different patches. In particular, Cobbold et al. (2022), Yurk and Cobbold (2018) developed the homogenisation theory that could handle discontinuities in population density at patch interfaces. Such discontinuities arise when individuals show patch preference when they reach a patch boundary (Maciel and Lutscher 2013; Schultz 1998).

Hence, the present work aims to understand how environmental heterogeneity affects PTW solutions in predator–prey systems, using asymptotic homogenisation. The PTW solutions of the homogenised model were analysed through the same approach as Sherratt et al. (2003): the application of a normal form transformation as an approximation to small amplitude PTWs. In Sect. 2, we present the homogenised predator–prey model originally presented by Cobbold et al. (2022) and apply normal formal analysis to study the PTW solutions. The approach provides conditions for stability of the small-amplitude PTW solutions. In Sect. 3, we analyse the effects of heterogeneity on both the period and amplitude of spatially uniform solutions and on the stability of small amplitude PTW solutions. In particular, we consider how PTW stability is affected by the interplay between heterogeneity in behaviour and movement.

## Methods

### The Patchy Landscape Model

The model describes a predator–prey system inhabiting an infinite one-dimensional landscape composed of a sequence (indexed by *i*) of two alternating patch types, 1 and 2, with lengths $l_1$ and $l_2$. The prey (*u*(*x*, *t*)) and predator (*v*(*x*, *t*)) population densities evolve in space (*x*) and time (*t*) according to the following reaction–diffusion equations

1 $$\begin{aligned}{} & {} \frac{\partial u}{\partial t} = D^u_i\frac{\partial ^2 u}{\partial x^2} + f_i(u,v),\nonumber \\ {}{} & {} \frac{\partial v}{\partial t} = D^v_i\frac{\partial ^2 v}{\partial x^2} + g_i(u,v), \quad \text {for} \quad x \in (x_{i-1}, x_i), \quad i \in \mathbb {Z}, \end{aligned}$$

where $x_i$ denote the boundaries of the patches.

**Fig. 1:**
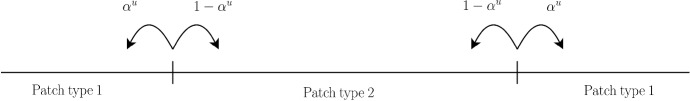
Example of the landscape structure composed of an infinite sequence of alternating patch types (1 and 2). The arrows represent the possibilities each prey individual has at the edge of the patch: it moves to patch type 1 with probability $\alpha ^u$ and to patch type 2 with probability $1-\alpha ^u$

Since Eq. (1) describes the dynamics in each patch, we refer to them as the *patch-level* equations. The diffusion rates of *u* and *v* inside patch *i* are constant within the patch and are denoted by $D^u_i$, $D^v_i$, respectively. If *i* is odd, $D^u_i = D^u_1$, if *i* is even, $D^u_i = D^u_2$, and analogously for *v*. The same holds for all the parameters indexed by *i*. When a prey individual encounters the interface between the two patches, we assume it chooses patch type 1 with probability $\alpha ^u$ and patch type 2 with probability $1-\alpha ^u$ (analogously, probabilities $\alpha ^v$ and $1-\alpha ^v$ for a predator individual). In Fig. 1, we illustrate the patch structure. The interface conditions for the prey population density (*u*) are the same as used by Yurk and Cobbold (2018), which are

2 $$\begin{aligned} \begin{aligned} (1-\xi ^u_i)D^u_{i+1}u(x^+_i,t)&= \xi ^u_iD^u_iu(x^-_i,t),\\ D^u_{i+1} \partial _x u(x^+_i,t)&= D^u_i \partial _x u(x^-_i,t), \end{aligned} \end{aligned}$$

where

3 $$\begin{aligned} \xi ^u_i = {\left\{ \begin{array}{ll} (1-\alpha ^u), &{}{}\quad \text{ if }\, i \,\text{ is } \text{ odd, }\\ \alpha ^u, &{}{}\quad \text{ if }\, i\, \text{ is } \text{ even, }\\ \end{array}\right. } \end{aligned}$$

and $x^+_i$, $x^-_i$ correspond to the right and left boundaries of patches *i* and $i+1$, respectively. Conditions (2) are derived from the assumption that the flux of individuals must be constant across any two patches. Different assumptions over individual behaviour at patch interface in the underlying random-walk model (Ovaskainen and Cornell 2003) lead to different interface conditions. Our model assumes scenario 3 from (Maciel and Lutscher 2013), in which individuals choose each patch with different probabilities as described, but keep the step size constant once inside the patches. An analogous set of conditions are assigned to the predator population density *v*.

We assume the reaction terms on each patch in equation (1) follow the Rosenzweig–MacArthur model (Rosenzweig and MacArthur 1963):

4 $$\begin{aligned} f_i(u,v) = (\lambda _i - \mu _i u ) u - \frac{a_i u v}{1 + a_i h_i u}, \quad g_i(u,v) = \gamma \frac{a_i u v}{1 + a_i h_i u} - m_i v. \end{aligned}$$

The Rosenzweig–MacArthur model is a predator–prey model with cyclic solutions for a well-known range of parameters (see Kot 2001). On each patch, the prey (*u*) grows logistically at a per capita rate $\lambda _i$, with the strength of the intraspecific competition denoted by $\mu _i$. The predator (*v*) attacks the prey at rate $a_i$ and the consumption of prey saturates (for fixed *v*) as *u* increases, as a consequence of each predator individual expending a nonzero amount of time (the handling time $h_i$) to consume each prey individual. The prey-to-predator conversion coefficient in this prey consumption is $\gamma$, and the predator is removed from the system at a per capita rate $m_i$ (mortality rate).

The assumption that the landscape is periodic may seem relatively restrictive. However, from a sufficiently large scale, many real landscapes can be seen as collection of repeating homogenous patches (Fitzgibbon et al. 2001). Therefore, the periodic landscape described above can be a good approximation to many real ecological environments (Garlick et al. 2011). The framework utilised in this paper (asymptotic homogenisation, Sect. 2.2) is valid in more general heterogeneous environments. The periodicity assumption is, however, mathematically convenient, providing simplifications that allows for analytical results.

### Asymptotic Homogenisation

The patchy landscape model described by equations (1), (4) and (2) is computationally expensive to numerically simulate for our case of interest, where we must consider large landscapes in order to observe PTW solutions. Asymptotic homogenisation (Holmes 2012) consists of averaging over the landscape heterogeneity, resulting in an approximated but much simpler version of the PDEs. We follow closely the work from Cobbold et al. (2022), where the homogenisation technique was developed for two-species reaction–diffusion systems on a patchy landscape, as presented in 2.1, with discontinuities at patch boundaries. The core underlying assumption for the asymptotic homogenisation to hold is that the patches are small enough that each individual is expected to go through a large number of patches during its lifetime. Through considerations involving the dynamic level of the populations (see Turchin 1998), it is possible to derive partial differential equations for the leading order of the power series expansion of the two populations’ densities (leading orders named *U* for the prey, *V* for the predator). Cobbold et al. (2022) obtain the set of homogenised equations for *U* and *V*,

5 $$\begin{aligned} \begin{aligned} \partial _t U&= \hat{D}^u \partial ^2_x U + \hat{f}(U,V) \\ \partial _t V&= \hat{D}^v \partial ^2_x V + \hat{g}(U,V), \end{aligned} \end{aligned}$$

with

6 $$\begin{aligned} \hat{f}(U,V) = \frac{f_1\Big (\frac{\rho ^u_1}{\langle \rho ^u\rangle }U,\frac{\rho ^v_1 }{\langle \rho ^v\rangle }V\Big )l_1 + f_2\Big (\frac{\rho ^u_2}{\langle \rho ^u\rangle }U,\frac{\rho ^v_2 }{\langle \rho ^v\rangle }V\Big )l_2}{l_1 + l_2} \end{aligned}$$

and $f_1, f_2$ given by Eq. (4). An analogous expression holds for *g* as an average of $g_1$ and $g_2$. The “averaging” brackets correspond to the formula

$$\begin{aligned} \langle \cdot \rangle = \frac{\cdot _1 l_1 + \cdot _2 l_2}{l_1 + l_2}. \end{aligned}$$

The parameter $\rho ^{u,v}_i$, given by

7 $$\begin{aligned} \rho ^{u,v}_i = \frac{1}{D^{u,v}_i\xi ^{u,v}_i}, \end{aligned}$$

is called the residence index. At each given location in space, it can be interpreted as a quantity, which is proportional to the steady-state population density at that location if this population was only subject to diffusion. It is a “relative measure of the average time that an organism spends between entering and leaving a unit area” Turchin (1998). In our case, these unit areas are the individual patches of the landscape, and therefore, the residence indices ($\rho ^{u,v}_1$, $\rho ^{u,v}_2$) are proportional to the average time each organism spends in each patch type. Equation (7) combines movement through diffusion ($D^{u,v}_i$) and behaviour through patch preference ($\xi ^{u,v}_1 = (1-\alpha ^{u,v})$ and $\xi ^{u,v}_2 = \alpha ^{u,v}$). If the residence index is large, it can be either because the individuals move slowly in that location or they have a high preference for that patch type.

The homogenised diffusion rates of Eq. (5) are given by:

8 $$\begin{aligned} \hat{D}^{u,v} = \langle \rho ^{u,v} \rangle ^{-1} \langle \xi ^{u,v} \rangle ^{-1}. \end{aligned}$$

By expanding Eq. (6), the analogous expression for $\hat{g}$ and redefining the parameters according to Table 1, we obtain the following expressions for the reaction terms of Eq. (5):

9 $$\begin{aligned} \begin{aligned} \hat{f}(U,V)&= (\hat{\lambda } - \hat{\mu } U)U - \Big [\frac{A_1 U}{1 + A_1 H_1 U} + \frac{A_2 U}{1 + A_2 H_2 U}\Big ]V, \\ \hat{g}(U,V)&= - \hat{m} V + \gamma \Big [\frac{A_1 U}{1 + A_1 H_1 U} + \frac{A_2 U}{1 + A_2 H_2 U}\Big ]V. \end{aligned} \end{aligned}$$

**Table 1:** Homogenised parameters from Eq. (9)

Parameter	Expression	Biological meaning
$\rho _1^{u,v}$	$\frac{1}{D_1^{u,v}(1-\alpha ^{u,v})}$	Residence index in patch type 1
$\rho _2^{u,v}$	$\frac{1}{D_2^{u,v}\alpha ^{u,v}}$	Residence index in patch type 2
$A_i$	$a_i \frac{\rho ^v_i}{\langle \rho ^v \rangle }\frac{\rho ^u_i}{\langle \rho ^u \rangle }\frac{l_i}{l_1 + l_2}$	Homogenised attack rate in patch type *i*
$H_i$	$h_i \Big (\frac{\rho ^v_i}{\langle \rho ^v \rangle }\Big )^{-1}\Big (\frac{l_i}{l_1+l_2}\Big )^{-1}$	Homogenised handling time in patch type *i*
$\hat{\lambda }$	$\lambda _1\Big (\frac{\rho _1^u}{\langle \rho ^u\rangle }\Big )\Big (\frac{l_1}{l_1+l_2}\Big ) + \lambda _2\Big (\frac{\rho _2^u}{\langle \rho ^u\rangle }\Big )\Big (\frac{l_2}{l_1+l_2}\Big )$	Averaged prey growth rate
$\hat{\mu }$	$\mu _1\Big (\frac{\rho _1^u}{\langle \rho ^u\rangle }\Big )^2\Big (\frac{l_1}{l_1+l_2}\Big ) + \mu _2\Big (\frac{\rho _2^u}{\langle \rho ^u\rangle }\Big )^2\Big (\frac{l_2}{l_1+l_2}\Big )$	Averaged prey intraspecific competition coefficient
$\hat{m}$	$m_1\Big (\frac{\rho _1^v}{\langle \rho ^v\rangle }\Big )\Big (\frac{l_1}{l_1+l_2}\Big ) + m_2\Big (\frac{\rho _2^v}{\langle \rho ^v\rangle }\Big )\Big (\frac{l_2}{l_1+l_2}\Big )$	Averaged predator mortality rate

The homogenised equations for *U* and *V* are similar to the ones at the patch level (1) for *u* and *v*, but due to spatial heterogeneity, have different reaction terms $\hat{f}$ and $\hat{g}$. Indeed, in the limiting case where all patch level parameters have the same values between patch types 1 and 2 and there is no patch preference (i.e. the limiting case of a homogeneous landscape), the reaction terms simplify and become identical to the Rosenzweig–MacArthur model (4). In this limiting case, our model equations match the ones studied by Sherratt et al. (2003).

It is worth highlighting that the factors $\Big (\frac{\rho _i^u}{\langle \rho ^u\rangle }\Big )\Big (\frac{l_i}{l_1+l_2}\Big )$ appearing as averaging weights in the expression for the homogenised parameters (Table 1) correspond to the relative amount of time that prey spends in type *i* patches if the system was only governed by diffusion (Cobbold et al. 2022). A completely analogous argument holds for the predator and the expression $\frac{\rho ^v_i}{\langle \rho ^v \rangle }\Big (\frac{l_i}{l_1+l_2}\Big )$.

In order to be able to directly compare with the work of Sherratt et al. (2003) and to simplify the analysis, we perform a non-dimensionalisation of (5) using the following rescaled parameters:

10 $$\begin{aligned} \begin{aligned}&T = \hat{\lambda } t, \qquad X = x\sqrt{\frac{\hat{\lambda }}{\hat{D}^u}}, \qquad h = \frac{\hat{\mu }}{\hat{\lambda }}U, \qquad p = \frac{\hat{\mu }}{\hat{\lambda }^2 H_1}V, \qquad C = A_1H_1\frac{\hat{\lambda }}{\hat{\mu }}, \\&B_1 = \frac{\hat{\lambda } H_1}{\gamma }, \qquad E_1 = \frac{\gamma }{\hat{m} H_1}, \qquad \beta = \frac{H_1}{H_2}, \qquad \delta = \frac{\hat{D}^v}{\hat{D}^u}, \qquad \eta = \frac{A_2H_2}{A_1H_1}. \end{aligned} \end{aligned}$$

The resulting rescaled equations are:

11 $$\begin{aligned} \begin{aligned} \partial _T h&= \partial ^2_X h + (1 - h)h - \Big [\frac{C h}{1 + C h} + \frac{\beta C \eta h}{1 + C \eta h}\Big ]p \\ \partial _T p&= \delta \partial ^2_X p - \frac{p}{E_1B_1} + \Big [\frac{C h}{B_1(1 + C h)} + \frac{\beta C \eta h}{B_1(1 + C \eta h)}\Big ]p, \end{aligned} \end{aligned}$$

where *h*(*X*, *T*) and *p*(*X*, *T*) correspond to the rescaled prey and predator densities, respectively. Note that if $\eta = 1$, Eq. (11) simplifies and the reaction terms have the same functional form as the Rosenzweig–MacArthur model.

### Normal Form Analysis

Equation (11) describes, with good approximation, the dynamics of the original patchy landscape model presented in Sect. 2.1, but are considerably easier to analyse and to numerically simulate. Equation (11) has up to 3 nonnegative spatially uniform equibria: extinction, prey-only, and coexistence. Cobbold et al. (2022) analysed the stability of these equilibria and showed that stable limit cycles exist. We are interested studying the region of parameter space where stable limit cycles occur. When our model is considered in the limiting case of a homogeneous landscape, the parameter used as a bifurcation parameter corresponds to the same used by Sherratt et al. (2003). Therefore, throughout this paper we use *C* as the bifurcation parameter to allow comparison between our work and Sherratt et al. (2003). There is a critical value of *C* ($C=C_{\textrm{crit}}(E_1,\eta ,\beta )$) at which the kinetics undergo a Hopf bifurcation, with stable limit cycles for values of *C* above $C_{\textrm{crit}}$. In the reaction–diffusion system, when $C>C_{\textrm{crit}}$, Eq. (11) can exhibit PTW solutions and standard analysis of these solutions is possible. We follow the script provided by the work of Sherratt et al. (2003), performing a reduction to normal form Guckenheimer and Holmes (2013). In the case of $\delta = 1$, the kinetics in Eq. (11) can be approximated by Hopf normal form giving equations of lambda-omega type (see, e.g. Murray 2001):

12 $$\begin{aligned} \begin{aligned} \frac{\partial \tilde{h}}{\partial t}&= \frac{\partial ^2 \tilde{h}}{\partial x^2} + (1 - r^2)\tilde{h} - (\omega _0 - \omega _1r^2)\tilde{p}, \\ \frac{\partial \tilde{p}}{\partial t}&= \frac{\partial ^2 \tilde{p}}{\partial x^2} + (\omega _0 - \omega _1r^2)\tilde{h} + (1 - r^2)\tilde{p}, \end{aligned} \end{aligned}$$

where $\tilde{h}$ and $\tilde{p}$ are nonlinear combinations of *h* and *p* (determined by the reduction to normal form (Sherratt et al. 2003) and $r^2 = \tilde{h}^2 + \tilde{p}^2$. With the aid of a computer algebra package, $\omega _0$ and $\omega _1$ can be written in terms of the homogenised parameters $E_1,B_1,\beta ,\eta ,C-C_{\textrm{crit}}$. The full *Mathematica* notebook for the normal form transformation and subsequent stability analysis are provided at the open-access repository (Andrade and Cobbold 2022). The calculations involved in finding $\omega _1(E_1,B_1, \eta ,\beta )$ were performed with *Mathematica* (Wolfram Research 2019). All plots produced in this paper were made with Python’s Matplotlib library (Hunter 2007).

The reduction to normal form consists of a nonlinear transformation of Eq. (11) into a simpler set of equations that approximate the original equations when *C* is close to $C_{\textrm{crit}}$, which we refer to as the small-amplitude regime. Thus, the stability of original PTW solutions can be inferred from the stability of the approximated PTW solutions that satisfy (12). Albeit limited to an approximation of small-amplitude solutions, the lambda-omega system provides insights into the dependence of the stability of PTW solutions on the ecological parameters. The work of Kopell and Howard (1973) shows that the lambda-omega system (Eq. 12) has a family of PTW solutions of the form:

13 $$\begin{aligned} \begin{aligned} \tilde{h}(x,t)&= R\sin \big [ (\omega _0 - \omega _1 R^2)t \pm (1 - R^2)x \big ], \\ \tilde{p}(x,t)&= R\cos \big [(\omega _0 - \omega _1 R^2)t \pm (1 - R^2)x \big ] \end{aligned} \end{aligned}$$

parameterised by *R*, the amplitude of the PTWs.

Typically PTWs are generated by either boundary conditions or population invasions. In order to study PTW stability analytically, we therefore consider a semi-infinite domain and we follow the approach from Sherratt et al. (2003), with zero-Dirichlet boundary condition at the origin. The asymptotic homogenisation and the reduction to normal form are still valid if the model described in Sect. 2.1 is semi-infinite. Sherratt et al. (2003) obtained a stability condition for the PTW solutions of the family (13) generated by Dirichlet boundary conditions if *C* is sufficiently close to $C_{\textrm{crit}}$. The derivation is overviewed in Appendix A. The condition is that a PTW solution (13) generated by a zero-Dirichlet boundary conditions at the origin is stable if and only if

14 $$\begin{aligned} |\omega _1| < 1.110468. \end{aligned}$$

Therefore, by transforming system (11) into its approximate version (12), we obtain an expression for $\omega _1$ in terms of the homogenised parameters $E_1,B_1, \eta$ and $\beta$. A different criterion holds for stability of PTWs generated by invasion (Sherratt 1998, 2001). Criterion (14) is then used to classify regions of the parameter space separating stable PTW and unstable PTW (spatiotemporal chaos). Figures 5, 7, 8, 9 and 10 in Sect. 3.2 are made using criterion (14) to classify each point of the paramater space as corresponding to a stable or an unstable PTW solution.

The reduction to normal form can only be applied to a particular form of Eq. (11), where $\delta = 1$. This corresponds to requiring the diffusion coefficients in equation (5) to be identical (see Eq. 8):

15 $$\begin{aligned} \delta = 1 \implies \hat{D}^u = \hat{D}^v. \end{aligned}$$

This constraint means that we have one less degree of freedom when choosing values for the parameters related to movement and space, $\alpha ^u,\alpha ^v, \rho _i^u, \rho _i^v,l_1,l_2$. For example, we can keep $\rho _1^v, \rho _2^v, l_1, l_2, \rho ^u_1, \alpha ^u,\alpha ^v$ as free parameters and (15) determines $\rho _2^u$ as

16 $$\begin{aligned} \rho ^u_2 = \frac{l_1+l_2}{l_2}\Big [\hat{D}^v \langle \alpha ^u\rangle ^{-1} - \frac{l_1}{l_1+l_2}\rho ^u_1\Big ]. \end{aligned}$$

We explore the biological implications of this constraint in the discussion.

## Results

**Fig. 2:**
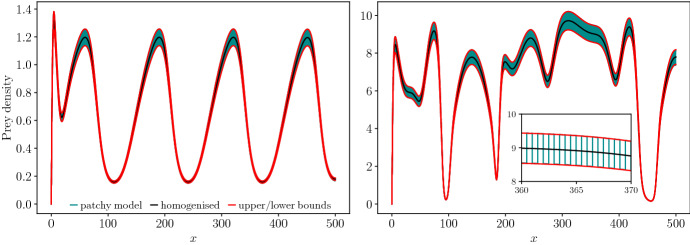
Comparison between the simulations of the patchy landscape model (blue, Eqs. 1, 2, 4) and the homogenised approximation (black, Eq. 5) prey (*U*) population densities. The upper and lower bounds are obtained through $$\rho _1^u U/\langle \rho ^u\rangle $$ and $$\rho _2^u U/\langle \rho ^u\rangle $$. Left (stable PTW): $$t = 450$$, $$m_1 = m_2 = 0.8, \mu _1 = \mu _2 = 5.0$$. Right (unstable PTW): $$t = 50$$, $$m_1 = m_2 = 1.5, \mu _1 = \mu _2 = 1.0$$. Initial conditions for the homogenised model are $$U(x,0) = 4(1+\sin (2\pi x/500))$$ for the prey and $$V(x,0) = 2(1+\sin (2\pi x/500))$$ for the predator. The corresponding prey initial conditions for the patchy landscape model were $$\rho _1^u U(x,0)/\langle \rho ^u\rangle $$ in patch type 1 and $$\rho _2^u U(x,0)/\langle \rho ^u\rangle $$ in patch type 2. Analogous conditions are used for the predator (*V*). Boundary conditions: zero-Dirichlet at $$x=0$$ and zero-flux at $$x=500$$. Other parameters: $$D^u_1 = 21, D^u_2 = 19, D^v_1 = 22, D^v_2 = 20$$, $$ l_1 = l_2 = 0.5$$, $$\alpha ^u = \alpha ^v = 0.5$$, $$a_1 = a_2 = 3.0, \gamma = 0.9, h_1 = h_2 = 0.5, \lambda _1 = \lambda _2 = 10.$$ The numerical simulations are carried out using the method described in Yurk and Cobbold (2018). The Strang splitting is applied, with diffusion terms implemented with Crank–Nicholson and fourth-order Runge–Kutta to update the kinetic step. For the patchy landscape model, derivatives that appear in the patch interface conditions were implemented using second-order forward or backward difference. Discretisation: $$\Delta t = 0.001, \Delta x = 0.1$$ (Color Figure Online)

Before analysing the behaviour of the homogenised model, we compare the PTW solutions of the homogenised equations (5) to solutions of the patchy landscape model (1). Figure 2 illustrates that the homogenised equations provide a very close approximation. In Fig. 2 (left), we illustrate a stable PTW moving in the positive *x*-direction. Changing parameter values leads to unstable PTW, giving rise to irregular oscillations and spatiotemporal chaos (Fig. 2 right). In the unstable PTW regime, for larger times the homogenised solution begins to deviate from the corresponding patchy landscape solution. This is a consequence of the spatiotemporal chaos associated with the unstable PTW, where small differences in the initial conditions due to the homogenisation approximation result in the long-term deviation of the two solutions (Sherratt et al. 1995; Sherratt 1996). The stable PTW solutions remain close for all times.

### Cyclic Properties of the Spatially Uniform Solutions

Before studying the PTW solutions of (11), we examine how patch heterogeneity affects the stable limit cycle solutions of the kinetic ordinary differential equations, which correspond to the spatially uniform equivalent of Eq. (11). At a particular value of $C = C_{\textrm{crit}}$, the non-trivial steady state undergoes a Hopf bifurcation and for $C > C_{\textrm{crit}}$ the non-trivial steady state $(h^*,p^*)$ is locally unstable and the kinetics have a stable limit cycle solution. $C_{\textrm{crit}}$ is determined by standard linear stability theory (Glendinning 1994). The Hopf bifurcation occurs when the eigenvalues, $\lambda ^*$, of the Jacobian matrix (*J*) of the kinetic ordinary differential equations evaluated at $(h^*,p^*)$ are purely imaginary, or equivalently when

$$\begin{aligned} \textrm{Tr}[J(h^*,p^*)] = 0. \end{aligned}$$

Solving this equation for *C* gives an expression for $C_{\textrm{crit}}$ in terms of $\eta , \beta , E_1, B_1$. The expression for $C_{\textrm{crit}}$ is algebraically messy (see section 1 of the Mathematica notebook (Andrade and Cobbold 2022)), but in the special case of $\eta =1$ it simplifies to

17 $$\begin{aligned} C_{\textrm{crit}} = \frac{E_1(\beta + 1)+1}{E_1(\beta + 1)-1}. \end{aligned}$$

Close to the Hopf bifurcation ($\textrm{Tr}[J(h^*,p^*)] \approx 0$), the temporal frequency of the limit cycle solutions is approximated by the imaginary part of $\lambda ^*$: $\sqrt{\det (J(h^*,p^*))}$. Therefore, the temporal period ($\tau$) of such solutions is approximated by:

$$\begin{aligned} \tau = \frac{2\pi }{\sqrt{\det (J(h^*,p^*))}}. \end{aligned}$$

At $\eta =1$, and $C=C_{\textrm{crit}}$ the temporal period simplifies to

18 $$\begin{aligned} \tau |_{C=C_{\textrm{crit}}} = 2\pi \sqrt{ B_1E_1\frac{(1+\beta )E_1+1}{(1+\beta )E_1-1}}. \end{aligned}$$

**Fig. 3:**
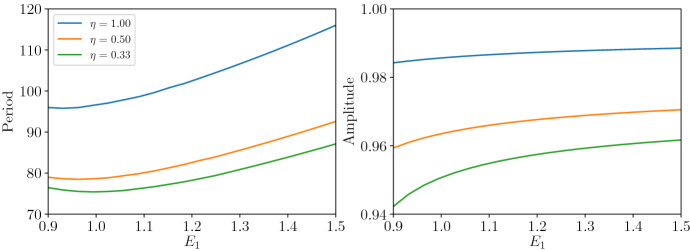
Period and amplitude of the spatially uniform prey temporal oscillations with respect to $E_1$ for different values of $\eta$. Other parameters $\beta = 1.0$, $B_1 = 10.0$, $C = 9.0$ (Color Figure Online)

In the special case of $\eta =1$, equation (18) demonstrates that for small amplitude cycles increasing $B_1$ increases period, while increasing $E_1$ has a nonlinear effect on period (Fig. 3). Our focus is on understanding the effects of heterogeneity, so we interpret how changes in $E_1$ and $B_1$ relate to patch differences. From expressions 10 we see that $E_1$ depends on $\hat{m}$, the patch averaged predator mortality rate. Thus, increasing $E_1$ can correspond to decreasing $\hat{m}$, or equivalently decreasing predator mortality rate on one or both patches. Since $\hat{m}$ is a weighted average of the patch mortality rates, the weights (the proportion of time spent on each patch) control which patch most strongly influences the value of $\hat{m}$.

In addition to letting $\eta =1$, if we assume predator mortality rate on both patches is the same ($m_1=m_2$) and also assume that prey handling time is constant across the landscape ($h_1=h_2$) then Eq. (18), and hence, cycle period is now independent of predator residence index (see Appendix B). By expressing $\eta$ in terms of dimensional parameters as

19 $$\begin{aligned} \eta = \frac{a_2 \rho ^u_2 h_2}{a_1\rho ^u_1 h_1}, \end{aligned}$$

the expression for $\eta$ gives us insights into the trade-offs between patch level parameters of the system. Consider an example in which predator attack rate varies between patch types. One might expect that if a predator spends more time in locations where attack rate is high this would increase total predator density and decrease prey density, impacting on population cycles, but this is not the case. Instead, the $\eta =1$ constraint ensures that any difference in patch attack rates is balanced by differences in prey residence index. Prey spend less time on patches with high attack rates, so predators would gain no advantage by spending more time in high attack rate patches and therefore cycle period will be independent of predator residence index.

**Fig. 4:**
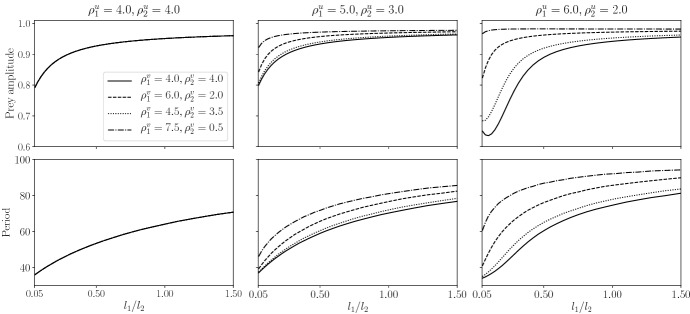
Amplitude (upper) and period (lower) of prey oscillations in the spatially uniform solution of (11) as a function of relative patch size for different values of predator residence index. Patch type 1 is the ‘good’ patch type ($\lambda _1 = 10$) and type 2, the ‘bad’ patch type ($\lambda _2 = 5$). Patch sizes $l_1, l_2$ satisfy $l_1 + l_2 = 1$. Left: $\rho _1^u = 4,\rho _2^u = 4$ (curves corresponding to each predator residence index pair are indistinguishable ). Centre: $\rho _1^u = 5,\rho _2^u = 3$. Right: $\rho _1^u = 6,\rho _2^u = 2$. Other parameters $h_1 = h_2 = 0.5, \gamma = 0.9,m_1 = m_2 = 1.0, \mu _1 = \mu _2 = 1.75, a_1 = a_2 = 3.0.$

Hence, $\eta =1$ presents a special case in which predator residence index does not affect population cycles, but prey movement does. In Fig. 3, we illustrate what happens as we relax this constraint on $\eta$. As $\eta$ increases both period and amplitude increases, and for $\eta \ne 1$ predator residence index *does* affect population cycles (Fig. 4). Higher values of $\eta$ correspond to larger patch differences in predator attack rate or handling time, which are not balanced by changes in prey movement. To fully understand these effects of heterogeneity, it is useful to consider the dimensional model (5) and the dimensional parameters (Table 1), allowing us to explicitly study the effects of prey and predator residence index, and patch size. The dimensional model also allows us to consider variation in patch prey growth rate, which we could not study in the non-dimensional model as average prey growth rate was used to scale time (see expressions 10).

In Fig. 4, we consider a scenario where prey growth rate on patch 1 (good patch) is greater than on patch 2 (bad patch), $\lambda _1 = 10 > \lambda _2 = 5$. We then vary the parameters associated with movement: $l_1/l_2$ (the relative size of patch 1 compared to patch 2) and prey and predator residence index. Predator amplitudes are shown in Appendix C. From the plots in Fig. 4, three main results should be highlighted.

Firstly, the overall trend shown in Fig. 4 is that the amplitudes and periods of the predator–prey cycles increase with the proportion of ‘good’ patch sizes (increasing $l_1/l_2$). This effect is particularly clear for small $l_1/l_2$ and can be explained by the fact that, if the good patches occupy a smaller proportion of the habitat, prey spend more time in the bad patches, where prey growth rate is lower, leading to smaller population cycle amplitudes. For larger $l_1/l_2$, our results suggest that both the period and amplitude saturate at a fixed value, which varies depending on the value of prey and predator residence indices. The only exception to the overall trend is shown at the rightmost amplitude plot ($\rho _1^u = 6.0, \rho _1^u = 2.0$). For small $l_1/l_2$, increasing $l_1/l_2$ can lead to a decrease in amplitude without an increase in period if $\rho ^v_1$ is close to $\rho ^v_2$. This exception can be explained in the following way. Since $\rho ^u_1$ is bigger than $\rho ^u_2$, prey spend more time in the good patches (patch 1). However, unless $\rho ^v_1$ is also considerably bigger than $\rho ^v_2$, predator do not spend as much time in patch type 1 as the prey, allowing prey to increase its population to larger values. The phenomenon of a decrease in amplitude with $l_1/l_2$ vanishes, as the proportion of good patches increases.

Secondly, we observe the effects of heterogeneity in predator residence index alone. In the centre and right plots of Fig. 4, the amplitude and periods of the cycles increase as we increase $\rho _1^v$ and decrease $\rho _2^v$ (solid to dotted to dashed to dashed-dotted lines). We obtain a similar qualitative result by just increasing $\rho _1^v$ or just decreasing $\rho _2^v$ (not shown). As the predator spends proportionally more time in the good patch (patch 1), where the prey also spends more time, there is an increase in the expected number of prey–predator encounters. Thus, predation is intensified and predator population grows to larger amplitudes. This causes prey oscillation amplitude to also be increased, with a corresponding increase in the period of the system.

Finally, we highlight the interplay between heterogeneity in prey and predator residence index. We find that the magnitude of the changes in period and amplitude is increased when there is heterogeneity in prey residence index (compare left and centre plots in Fig. 4). Moreover, the greater the heterogeneity in prey residence index, the greater the effect of heterogeneity in predator residence index (compare centre and right plots). In particular, if $\rho ^u_1 = \rho ^u_2$, predator residence index has no effect on period and amplitude of prey cycles. This indicates that the time populations spend in each patch type are an important factor in determining the properties of the population cycles.

### Stability of Periodic Travelling Waves

#### Stability Boundaries of the Non-dimensional Homogenised Model

**Fig. 5:**
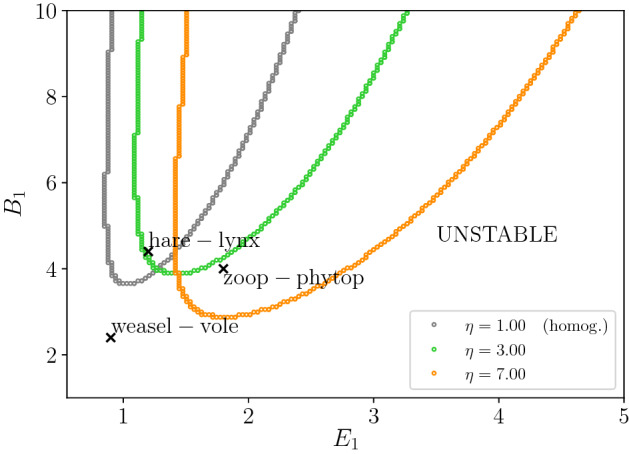
Stability boundaries in terms of the dimensionless parameters $E_1$, $B_1$ for different $\eta$, for $\beta = 1.0$ when *C* is close to $C_{\textrm{crit}}$. The areas above the curves correspond to parameter regions where the PTW solutions are stable. The regions below each curve correspond to unstable PTW solutions. We plot with respect to $B_1$ (prey/predator maximum birth rate) and $E_1$ (predator birth/death rate) so that in the limiting case ($\eta = 1.0$ corresponding to spatial homogeneity) we obtain the corresponding stability boundary discussed by Sherratt et al. (2003). The crosses denote the parameter sets given by Sherratt (2001) for three example predator–prey systems: hare-lynx, zooplankton-phytoplankton and weasel-vole (Color Figure Online)

In this section, we consider the stability of the PTW solutions of Eq. (11). We show how heterogeneity could affect the stability of real biological systems exhibiting PTWs. In Fig. 5, we illustrate the stability regions for different values of $\eta$ with respect to $E_1,B_1$, in order to directly compare to the results from Sherratt et al. (2003). The plot illustrates that stable PTWs are present for intermediate values of predator birth/death rates ($E_1$) and when the ratio of prey and predator maximum birth rate ($B_1$) is sufficiently high. Increasing the value of $\eta$ (moving from the grey to the green and yellow curves) corresponds to increasing landscape heterogeneity by making the two patch types more distinct, either through increasing differences in prey residence index or through increasing differences in attack rate. If we consider the three biological examples discussed by Sherratt (2001), we find that increasing $\eta$ shifts the hare-lynx system from a stable to an unstable PTW, and the zooplankton–phytoplankton system shifts from the unstable to the stable PTW region. On the other hand, the weasel-vole is predicted to remain in the unstable regime. This suggests that landscape heterogeneity could influence the dynamics observed in natural systems.

**Fig. 6:**
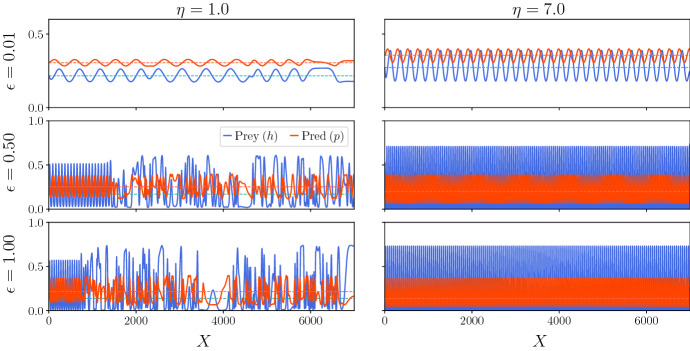
Numerical simulations of the homogenised equations (11) for the zooplankton–phytoplankton system parameter values ($E_1 = 1.80, B_1 = 4.0$), $\eta = 1.0$ (left plots) and $\eta = 7.0$ (right plots) and different values of the bifurcation parameter *C* with respect to its critical value $C_{\textrm{crit}}$: $\epsilon = C-C_{\textrm{crit}} = 0.01$ (upper plots), 0.5 (centre plots), 1.0 (lower plots). The solutions are plotted at $T =$ 500,000. The Strang splitting is applied, with diffusion terms implemented with Crank–Nicholson and fourth-order Runge–Kutta to update the kinetic step. Discretisation: $\Delta T = 0.1, \Delta X = 1$. Boundary conditions: Dirichlet $(h,p) = (h^*,p^*)$ at $X=0$ (steady state, dashed lines) and zero flux at $X = L = 7000$. Initial conditions for prey (*h*) and predator (*p*) were step functions with constant random values between 0 and 1 inside each of 100 equal size subdivisions of the full domain. Other parameters: $\beta = 1$, $\delta = 1$ (Color Figure Online)

Such predictions are valid for small amplitude solutions. However, ecologists are generally interested in larger-amplitude PTWs. In Fig. 6, we illustrate the shift in stability with numerical simulations of the homogenised equations (11) for three amplitude values, measured in terms of the difference between the bifurcation parameter *C* and its critical value $C_{\textrm{crit}}$ ($\epsilon = C - C_{\textrm{crit}} = 0.01, 0.5, 1.0$: upper, centre and lower plots, respectively). We consider the parameters of the zooplankton–phytoplankton system ($E_1 = 1.8$, $B_1 = 4.0$ (Sherratt 2001)) for two different values of $\eta :1$ (left plots) and 7 (right plots). We find that as predicted from Fig. 5, the solutions for $E_1 = 1.8, B_1 = 4.0$ are unstable for $\eta = 1$ and stable for $\eta = 7$. Moreover, such result holds both for smaller ($\epsilon = 0.01$) and for larger amplitudes ($\epsilon = 0.5,1.0$).

However, the stability shift predicted for small amplitude does not necessarily hold for larger amplitudes for all points of the $(E_1,B_1)$-parameter space. In fact, the stability of larger-amplitude PTW solutions is still an open problem in the literature. Moreover, the direct classification of PTW stability through numerical integrations is additionally challenging since simulating Eq. (11) for $E_1, B_1$ values close to the stability boundaries can require longer domains and simulation times to differentiate between stable and unstable solutions. This is illustrated in Appendix D, where it is shown plots for the numerical simulations for $E_1,B_1$ of the hare-lynx and weasel-vole systems from Fig. 5.

#### Heterogeneity in Prey Residence Index

The remainder of the results section examines PTW solutions in terms of the parameters of the dimensional model (5). This allows us to isolate the effects of various sources of landscape heterogeneity (e.g. patch variation in movement, attack rates, etc.) that cannot be teased apart by looking at the non-dimensional model. We study the ($\hat{\lambda }-\hat{m}$)-parameter space, as these parameters appear in the expressions for $E_1$ and $B_1$, respectively. Throughout the results section, we consider the case where prey growth rates and predator mortalities are identical between the two patches ($\lambda _1 = \lambda _2$ and $m_1 = m_2$), so that $\hat{\lambda }$ and $\hat{m}$ do not depend on $\rho ^u_i$ and $\rho ^v_i$. Therefore, we assume the only parameters which vary between patches are prey residence index ($\rho ^u_i$), predator residence index ($\rho ^v_i$) and attack rate ($a_i$). The normal form analysis constrains us to select prey and predator residence indices in such a way that the ratio of the homogenised predator and prey diffusion coefficients is 1 (Eq. 15). For example, if $\hat{D}^v$ is fixed, increasingly higher values of $\rho _1^u$ must be accompanied of, e.g. decreasingly smaller values of $\rho ^u_2$ in such a way that $\hat{D}^u$ has the same value for every pair $(\rho ^u_1, \rho ^u_2)$ considered.

**Fig. 7:**
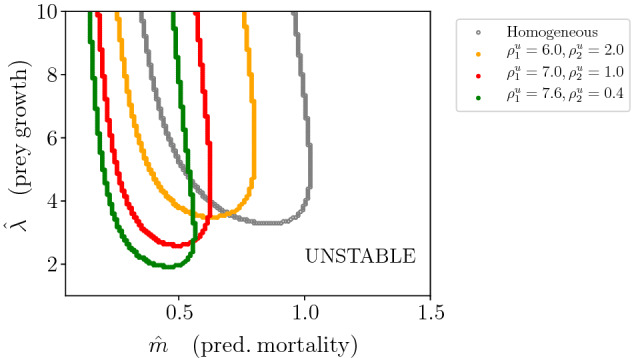
Stability boundaries for different values of prey residence index (coloured curves) in terms of the per capita prey growth rate ($\hat{\lambda }$) and the per capita predator mortality rate ($\hat{m}$), when the system is close to the Hopf bifurcation (when *C* is close to $C_{\textrm{crit}}$). The homogeneous boundary corresponds to do patch variation in prey residence index ($\rho ^u_1 = 4.0, \rho ^u_2 = 4.0$). The areas above the curves correspond to regions of parameters where the PTW solutions are stable, whereas the areas below the curves correspond to regions where the PTW solutions are unstable. $\lambda _1 = \lambda _2$ and $m_1 = m_2$, hence $\hat{\lambda }$ and $\hat{m}$ do not depend on $\rho ^{v,u}_i$. The other parameters are $\rho ^v_1 = \rho ^v_2 = 4.0, a_1 = a_2 = 0.5, h_1 = h_2 = 0.5, \gamma = 0.9, l_1 = l_2 = 0.5.$ (Color Figure Online)

In this subsection, we consider the effects of prey residence index alone. In Fig. 7, it is shown that the PTW stability boundary shifts towards smaller predator mortalities and becomes narrower as heterogeneity in prey residence increases (increasing $\frac{\rho ^u_1}{\rho ^u_2}$). The shift can be interpreted in the following way: increasing $\frac{\rho ^u_1}{\rho ^u_2}$ means that the prey tend to spend more time in patch type 1. The predator, whose residence index is unchanged, encounters proportionally less prey in patch type 2. This causes lower predator growth rate across the landscape. Hence, for stable periodic travelling waves to be obtained, the predator needs to survive long enough in order to spend a sufficient amount of time in the locations where the prey has a larger residence index (patch type 1). Longer predator survival corresponds to smaller values of $\hat{m}$. Therefore, the stability boundaries shift towards smaller values of predator mortality rate as $\frac{\rho ^u_1}{\rho ^u_2}$ increases.

#### Heterogeneity of Attack Rate

**Fig. 8:**
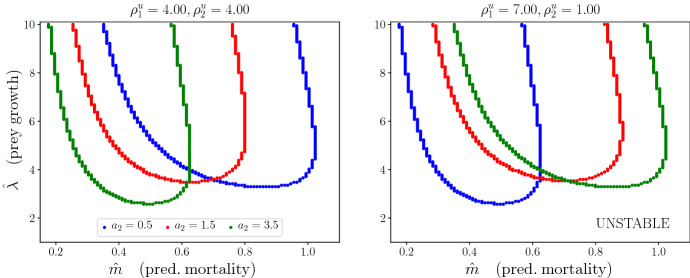
The effect of varying the attack rate on patch type 2 ($a_2$, coloured curves) in the stability boundaries for different combinations of prey residence index ($\rho ^u_2,\rho ^u_1$). Plots with respect to per capita prey growth rate ($\hat{\lambda }$) and the per capita predator mortality rate ($\hat{m}$), when the system is close to the Hopf bifurcation (when *C* is close to $C_{\textrm{crit}}$). The areas above the curves correspond to regions of parameters where the PTW solutions are stable, whereas the areas below the curves correspond to regions where the solutions are unstable. $\lambda _1 = \lambda _2$ and $m_1 = m_2$; hence, $\hat{\lambda }$ and $\hat{m}$ do not depend on $\rho ^{v,u}_i$. The other parameters are $\rho ^v_1 = \rho ^v_2 = 4.0, a_1 = 0.5, h_1 = h_2 = 0.5, \gamma = 0.9, l_1 = l_2 = 0.5.$ (Color Figure Online)

Figure 8 shows stability boundaries for varying values of attack rate in patch type 2 (coloured curves). The main result is that heterogeneity in prey residence index can compensate for the effects of heterogeneity in attack rate. This is precisely the trade-off discussed in Sect. 3.1. The different curves in Fig. 8 correspond to different values of attack rates in patch type 2 ($a_2$), whereas $a_1$ is fixed. In the left plot, prey residence index is the same on both patch types. In the right plot, prey residence index is larger in patch type 1 and smaller in patch type 2. If we increase prey residence index in patch type 1 enough, it is possible to counter act the effect of heterogeneity in attack rate, as we show in the right plot, where the green curve corresponds exactly to the homogeneous landscape stability boundary in Fig. 7 since $a_2$,$a_1$,$\rho ^u_1$,$\rho ^u_2$ are such that $\eta = 1$.

The opposing effects of heterogeneity in attack rate and prey residence index can be interpreted as follows: if the predator attacks disproportionally more in one of the patch types, it has an equivalent effect to the prey occupying the other patch type proportionally more, where it is being preyed upon proportionally less often.

#### Heterogeneity of Predator and Prey Residence Indices

**Fig. 9:**
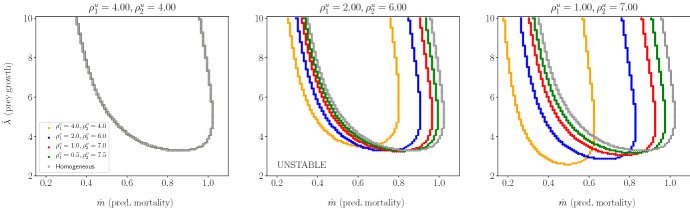
The effect of varying predator residence index (($\rho ^v_2,\rho ^v_1$), coloured curves) on the stability boundaries for different combinations of prey residence index ($\rho ^u_2,\rho ^u_1$). Plots with respect to per capita prey growth rate ($\hat{\lambda }$) and the per capita predator mortality rate ($\hat{m}$), when the system is close to the Hopf bifurcation (when *C* is close to $C_{\textrm{crit}}$). The areas above the curves correspond to regions of parameters where the PTW solutions are stable, whereas the areas below the curves correspond to regions where the solutions are unstable. $\lambda _1 = \lambda _2$ and $m_1 = m_2$, hence $\hat{\lambda }$ and $\hat{m}$ do not depend on $\rho ^{v,u}_i$. The other parameters are $a_1 = a_2 = 0.5, h_1 = h_2 = 0.5, \gamma = 0.9, l_1 = l_2 = 0.5.$ (Color Figure Online)

In Fig. 9, we show stability boundaries for varying predator residence indices (coloured curves) in patch type 2. The overall result is that strong heterogeneity in prey residence index increases the effect that heterogeneity in predator residence index has upon the location of the stability boundary. As before, prey and predator residence indices are selected in such a way that constraint (15) is satisfied. Different plots correspond to different values of prey residence index. The leftmost plot illustrates that predator residence index has no effect on stability if prey residence index does not vary with patch type (other parameters being fixed). This is consistent with the result from Sect. 3.1. As we move from the left to right plot in Fig. 9, increasing heterogeneity in prey residence index, we notice an increased sensitivity to heterogeneity in predator residence index. The curves for different $\rho _1^v,\rho _2^v$ pairs move further away from the $\rho _1^v = \rho _2^v$ curve as $\rho ^u_2/\rho ^u_1$ gets large. We saw a similar interaction between the effects of prey and predator residence indices in Sect. 3.1.

In the particular case where prey tend to spend equal time in both patches (Fig. 9, leftmost plot), the stability of the PTW solutions is unaffected by heterogeneity in predator residence index. This last result is explained from the fact that $\omega _1$ does not depend on $\rho ^v_i$ (see Appendix B) when $\eta = 1$ and predator mortalities and handling times are identical between the two patches ($m_1 = m_2$, $h_1 = h_2$).

**Fig. 10:**
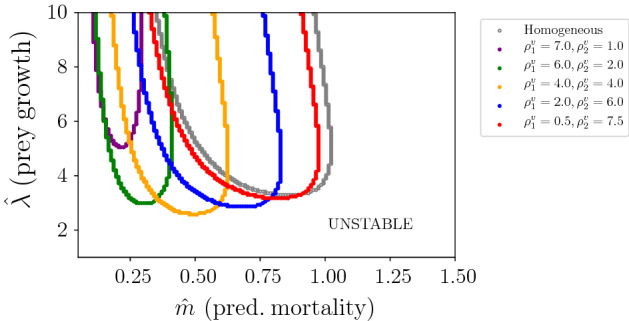
Stability boundaries for different levels of heterogeneity on predator residence index in terms of the per capita prey growth rate ($\hat{\lambda }$) and the per capita predator mortality rate ($\hat{m}$), when the system is close to the Hopf bifurcation (when *C* is close to $C_{\textrm{crit}}$). The areas above the curves correspond to regions of parameters where the PTW solutions are stable, whereas the areas below the curves correspond to regions where the solutions are unstable. $\lambda _1 = \lambda _2$ and $m_1 = m_2$, hence $\hat{\lambda }$ and $\hat{m}$ do not depend on $\rho ^{v,u}_i$. The other parameters are $\rho ^u_1 = \rho ^u_2 = 4.0, a_1 = 0.5, a_2 = 3.5, h_1 = h_2 = 0.5, \gamma = 0.9, l_1 = l_2 = 0.5.$ (Color Figure Online)

In Fig. 10, we fix prey residence index so that it does not vary with patch type and attack rate is chosen to be larger in patch type 2 ($a_2>a_1$). Here we highlight how heterogeneity in predator residence index interplays with heterogeneity in attack rate and predator mortality. In particular, heterogeneity in residence index can act to compensate or intensify the shift caused by heterogeneity in attack rate. The efficiency of the predator in the landscape depends on where it spends its time, how frequently it attacks prey in each location and how long it is alive. Hence, we see a trade-off of these quantities when we examine PTW stability boundaries. Decreasing $\rho ^v_2/\rho ^v_1$ (from yellow to green to purple curves), so that predators spend more time in patches of type 1 where attack rates are low. This causes the stability region to shift to the left and become narrower, meaning that the predator needs to have smaller mortality rate to ensure stable PTWs and that stable PTWs only exist in a small region of the parameter space. The increased tendency of predator to occupy patch 1, where the attack rate is smaller, acts to the reduce overall effectiveness of the predator. The reduced effectiveness can be compensated for by lower predator mortality rates. The same predator efficacy could be obtained by a long-lived predator with a low prey attack rate, or a short-lived predator with a high attack rate. This last claim is supported by the shift from yellow to blue and red curves as we increase $\rho ^v_2/\rho ^v_1$. As the predators tend to occupy patch type 2 more frequently, increased predator mortality rates can maintain PTW stability (right shifting curves). In summary, stability can be controlled by patch differences in predator residence index.

## Discussion

PTWs are often observed in ecological systems (Sherratt 2008) and were the focus of works that discussed their potential drivers (Kaitala and Ranta 1998; Petrovskii and Malchow 1999). As PTWs are an inherently spatial phenomena, it is natural to ask what are the implications of landscape alterations for such spatiotemporal dynamics. One way by which such alterations can happen is through the introduction of heterogeneity, which is expected to have an effect on ecological systems (Fahrig 2003). Environmental heterogeneity is known to affect PTWs (Shigesada et al. 1986; Sherratt et al. 2003; Kay and Sherratt 2000), but there is a lack of studies assessing the effects of heterogeneity that manifests through species movement and behaviour. Theoretical research can help to elucidate such effects and help identify when we expect to observe stable PTWs.

Our approach is the application of asymptotic homogenisation to study PTW solutions in a predator–prey model on a patchy landscape. Through a reduction to normal form, the stability of the PTW solutions is then analysed in terms of heterogeneity of residence index and attack rate. Our main result is that heterogeneity in prey and predator residence indices alone can shift biological systems from stable to unstable PTW regime and vice versa. This has implications for land management. For example, deforestation can change the proportion of forested to open areas. Many species show preference for forested habitats (Bélisle and Desrochers 2002), resulting in individuals spending more time in forested patches of land. This shift in residence index can be accompanied by a shift in the stability of PTWs. A given predator–prey exhibiting PTWs could see these destabilised resulting in irregular spatiotemporal oscillations. For the well-studied lynx-hare system (Turchin 2003; Krebs et al. 2018), our results suggest that introduction of environmental heterogeneity, such that hare spend more time in some locations than in others while lynx movement is unchanged, could result in the system being less likely to exhibit stable PTWs.

More generally, we contribute to the body of theoretical research that investigates the effects of environmental heterogeneity on predator–prey interactions (Ryall and Fahrig 2006; Stone and He 2007; Vitense et al. 2016). We showed that if prey spend disproportionally more time in one patch, the PTW stability boundary curve shift towards smaller predator mortalities. Such a shift can be reversed if the predator either spends more time or attacks more prey in the patches of high prey residence index. Our findings then illustrate the importance of the residence index as a useful tool to understand landscape-level phenomena. Since the residence index combines the effects of patch preference and diffusion, we stress the importance of patch-level properties on landscape-level patterns. The significance of patch properties has also been echoed by others, including Maciel and Lutscher (2013), who found that increased preference for a suitable (good) patch over an inhospitable (bad) surrounding patch decreased the minimal size of the good patch required for survival of the focal species.

The findings we obtain can be related to the work of Kay and Sherratt (2000) who considered PTWs generated by invasion on a finite domain. They showed that sufficient spatial noise in the kinetic parameters can allow the persistence of PTWs. However, if the noise is present at higher levels, irregular oscillations persist. In our work, we show that the heterogeneity in movement and individual behaviour can have complex effects on PTW stability, either fostering or suppressing stability of PTWs, depending on the values of the other kinetic parameters.

We show that the effects of predator residence index heterogeneity on PTW stability are increased as we increase the strength of heterogeneity in prey residence index. Our results suggest that a key factor affecting cyclic phenomena in general is the difference between the time prey and predator spend in each location. We argue that prey residence index should receive special attention in studies aiming to track and monitor predator–prey systems potentially exhibiting PTWs. We predict that if prey spend time relatively homogeneously across the landscape, heterogeneity in predator residence index should have a relatively small effect on the stability of the PTWs.

When considering the temporal cycles of spatially uniform solutions, we found a general increase in cycle period and amplitude with the relative good patch size. This result agrees with the overall trend obtained by previous reaction–diffusion predator–prey models (Maciel and Kraenkel 2014; Strohm and Tyson 2009). In a study involving coarse grain heterogeneity Gauduchon et al. (2013) showed that cycle amplitude in the middle of the patch decreases with predator patch preference for the good patch in the Rosenzweig–MacArthur model, but increases with predator patch preference in the May model. Despite our patch level equations having Rosenzweig–MacArthur reaction terms, our model shows a similar result to the May model studied by Gauduchon et al. (2013). The reasons for this mismatch remain unclear, but likely relates to the differing effects of coarser and finer grain heterogeneity on the Rosenzweig–MacArthur model.

Our analysis of the stability of the PTWs is constrained to the case where the homogenised prey and predator diffusion rates were equal. In biological systems, predator diffusion is typically larger than prey diffusion, so the $$\delta =1$$ constraint may appear overly constrictive. Indeed, the ratio between prey and predator diffusion is known to have an effect on the stability boundary of the PTWs in homogeneous systems (Smith and Sherratt 2007; Bennett and Sherratt 2017). However, $$\delta = 1$$ can be satisfied while still allowing predator diffusion to be larger than prey diffusion at the patch level. For example, we can consider a scenario where the predator moves faster than the prey in both patches (namely, $$D_1^u < D_1^v$$ and $$D_2^u < D_2^v)$$. This can be achieved while keeping $$\delta = 1$$ if prey and predator prefer different patch types (see Appendix E). Differences in prey and predator habitat preference have been observed in the Vicuñas-Puma system (Smith et al. 2019) when prey chose habitat to avoid being attacked. Hence, while the $$\delta = 1$$ constraint is restrictive it still allows the study biologically plausible scenarios. Future research with the use of alternative techniques would help elucidate the effects of relaxing the $$\delta = 1$$ constraint. Among such techniques, we highlight numerical continuation, which can be used to numerically determine the essential spectra of reaction–diffusion operators (Rademacher et al. 2007). The stability of PTWs solutions can then be assessed through the study of such spectra (Sherratt 2013).

Our results are restricted to the scenario where asymptotic homogenisation is valid. Namely, when patch sizes are small compared to species diffusion. Other approaches would be necessary to investigate the effects of coarser grain heterogeneity and with larger patch sizes (e.g. as done by Gauduchon et al. (2013), Maciel and Kraenkel (2014), and Cobbold and Lutscher (2014)). However, even under this limitation, we are able to show that heterogeneity in movement and behaviour is able to affect PTWs in complex ways.

The greater part of our study is performed in the small-amplitude approximation. In general, ecologists are interested in larger-amplitude population cycles. Merchant and Nagata (2010), Merchant and Nagata (2015) developed approaches to study particular PTWs solutions of predator–prey systems further away from the Hopf bifurcation. However, the analytical study of general PTW solutions for larger-amplitude oscillations remains an open problem in the literature and is therefore a possible step for future work. A natural question is whether our conclusions involving the effects of heterogeneity in movement and behaviour on PTW stability continue to hold for larger-amplitude PTWs. Nonetheless, the investigation of small amplitude is still used in the literature (Bennett and Sherratt 2017, 2019) as it provides useful analytical insights and limiting case predictions. Moreover, it produces a valuable framework to numerically investigate larger amplitude scenarios and a guideline to explore the parameter space through other non-analytical methods (e.g. numerical continuation).

Furthermore, another topic that deserves systematic study is the exploration of the effects of heterogeneity on PTW speed. Predicting the speed at which populations travel across the landscape is naturally a useful tool for monitoring and conservation studies but was beyond the scope of this work. As argued by Cobbold et al. (2022) and through our results, fine scale heterogeneity can lead to significantly different predictions from those found in a homogeneous environment. Therefore, one may expect that heterogeneity will also affect PTW speed.

In summary, our results shed light on factors affecting the stability of PTWs. In particular, we have shown that movement and behaviour in a heterogeneous environment can change the stability of PTWs in regions of parameter space where real-world examples of predator–prey dynamics can be found. We highlight that the strength of spatial variation in predator and prey residence indices can have a strong influence on PTW stability. Hence, future empirical research aiming to predict the effects of environmental heterogeneity (e.g. caused by human activity or climate change) on population spatiotemporal oscillations should take into consideration its repercussions on variation of individual movement and behaviour across the landscape.

## Figures and Tables

**Fig. 11:**
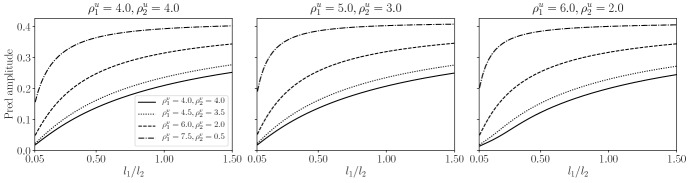
Amplitude of the spatially uniform predator oscillations as a function of the relative patch size for different values of predator residence index. Patch type 1 is the ‘good’ patch type ($\lambda _1 = 10$) and type 2, the ‘bad’ patch type ($\lambda _2 = 5$). $l_1, l_2$ satisfy $l_1 + l_2 = 1$. Left: $\rho _1^u = 4,\rho _2^u = 4$. Centre: $\rho _1^u = 5,\rho _2^u = 3$. Right: $\rho _1^u = 6,\rho _2^u = 2$. Other parameters $h_1 = h_2 = 0.5, \gamma = 0.9,m_1 = m_2 = 1.0, \mu _1 = \mu _2 = 1.75, a_1 = a_2 = 3.0.$

**Fig. 12:**
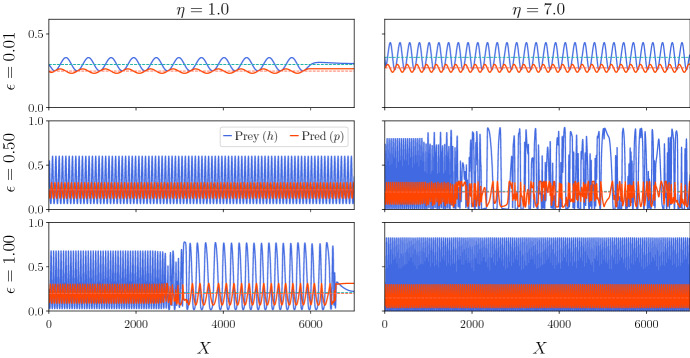
Numerical simulations of the homogenised equations (11) for the demographic parameters of the hare-lynx ($E_1 = 1.2, B_1 = 4.4$) system from Fig. 5. We consider the $\eta = 1.0$ (left plots) and $\eta = 7.0$ (right plots) cases, for different values of the bifurcation parameter *C* with respect to its critical value $C_{\textrm{crit}}$: $\epsilon = C-C_{\textrm{crit}} = 0.01$ (upper plots), 0.5 (centre plots), 1.0 (lower plots). The solutions are plotted at $T =$ 500,000. The same initial conditions, numerical scheme and discretisation from Fig. 6 were used. Boundary conditions: Dirichlet $(h,p) = (h^*,p^*)$ at $X=0$ (steady state-dashed lines) and zero-flux at $X = L = 7000$. Other parameters: $\beta = 1$, $\delta = 1$ (Color Figure Online)

**Fig. 13:**
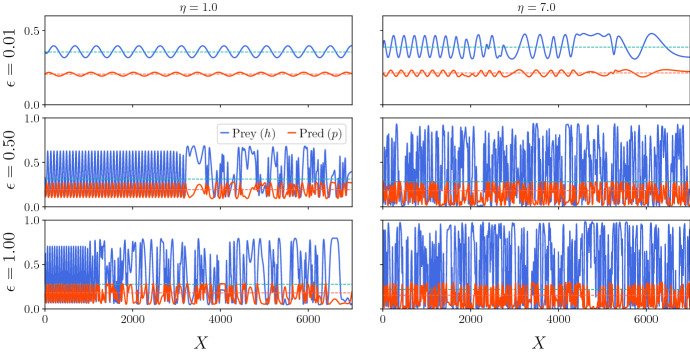
Numerical simulations of the homogenised equations (11) for the demographic parameters of the weasel-vole system ($E_1 = 0.9, B_1 = 2.4$) system from Fig. 5. We consider the $\eta = 1.0$ (left plots) and $\eta = 7.0$ (right plots) cases, for different values of the bifurcation parameter *C* with respect to its critical value $C_{\textrm{crit}}$: $\epsilon = C-C_{\textrm{crit}} = 0.01$ (upper plots), 0.5 (centre plots), 1.0 (lower plots). The solutions are plotted at $T =$ 500,000. The same initial conditions, numerical scheme and discretisation from Fig. 6 were used. Boundary conditions: Dirichlet $(h,p) = (h^*,p^*)$ at $X=0$ (steady state—dashed lines) and zero-flux at $X = L = 7000$. Other parameters: $\beta = 1$, $\delta = 1$ (Color Figure Online)

**Fig. 14:**
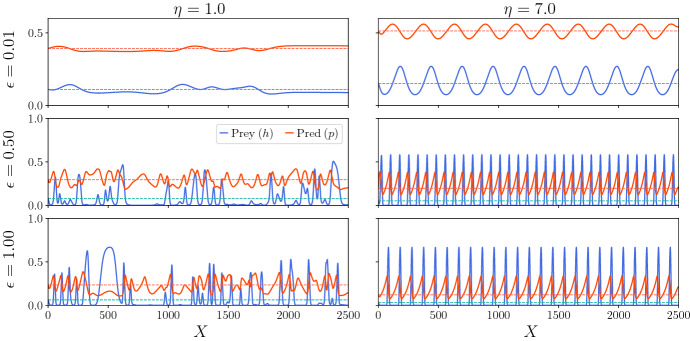
Numerical simulations of the homogenised equations (11) for $E_1 = 4.0, B_1 = 10.0$. We consider the $\eta = 1.0$ (left plots) and $\eta = 7.0$ (right plots) cases, for different values of the bifurcation parameter *C* with respect to its critical value $C_{\textrm{crit}}$: $\epsilon = C-C_{\textrm{crit}} = 0.01$ (upper plots), 0.5 (centre plots), 1.0 (lower plots). The solutions are plotted at $T =$ 500,000. The same initial conditions, numerical scheme and discretisation from Fig. 6 were used. Boundary conditions: Dirichlet $(h,p) = (h^*,p^*)$ at $X=0$ (steady state—dashed lines) and zero-flux at $X = L = 2500$. Other parameters: $\beta = 1$, $\delta = 1$ (Color Figure Online)

## Appendix A: Stability Criterion

In this work, we considered the stability of PTWs in terms of the heterogeneity in parameters determining movement and behaviour of prey and predator throughout the landscape. In this section, we review the mathematical background developed by Sherratt et al. (2003) leading to the criterion (14) for determining PTW stability.

Kopell and Howard (1973) have shown that the PTW solutions (13) are stable if and only if

20 $$\begin{aligned} R > \Big (\frac{2+2\omega _1^2}{3+2\omega _1^2}\Big )^\frac{1}{2}, \end{aligned}$$

where *R* corresponds to the amplitude of the PTW. Sherratt (2003) then showed that the boundary condition $\tilde{h}=\tilde{p}=0$ at $x=0$ on Eq. (12) generates a particular solution of the family (13), in which *R* takes the value of

21 $$\begin{aligned} R_0 = \left[ \frac{1}{2}\left( 1+\sqrt{1+\frac{8}{9}\omega _1^2}\right) \right] ^{-1/2}. \end{aligned}$$

Solutions under boundary condition $\tilde{h}=\tilde{p}=0$ at $x=0$ correspond to solutions of Eq. (11) under Dirichlet boundary conditions $h = h^*$ and $p = p^*$ at $X=0$, where $(h^*,p^*)$ is the steady-state solution. Therefore, combining (20) and (21), stability condition for the PTW solutions of Eq. (11) under Dirichlet boundary conditions $h = h^*$ and $p = p^*$ at $X=0$ is

$$\begin{aligned} R_0 > \Big (\frac{2+2\omega _1^2}{3+2\omega _1^2}\Big )^\frac{1}{2}, \end{aligned}$$

which can be manipulated to obtain $|\omega _1| < 1.110468$.

Finally, Sherratt et al. (2003) give solid evidence that such condition is valid for general Dirichlet boundary conditions at $X = 0$ provided the bifurcation parameter is sufficiently close to its critical value. Therefore, condition (14) determines the stability of solutions of Eq. (11) under zero-Dirichlet boundary condition at $X=0$.

## Appendix B: Stability Results in the Case of $\eta = 1$

We note that if $\eta = 1$, $C_{\textrm{crit}}$ (Eq. 17) and $\det (J(h^*, p^*))$ (see Eq. 18) are composed of the expressions $E_1(1+\beta )$ and $E_1 B_1$. Expressing these using the dimensional parameters listed in Table 1, we obtain

22 $$\begin{aligned} E_1(1+\beta ) = \frac{\gamma }{\hat{m}}\Big (\frac{1}{H_1} + \frac{1}{H_2}\Big ) \end{aligned}$$

and

23 $$\begin{aligned} E_1B_1 = \frac{1}{\hat{m}}. \end{aligned}$$

Through direct algebra, it is immediate to verify that if predator mortality rate and prey handling time are constant across the landscape (namely, $h_1 = h_2 = h$ and $m_1 = m_2 = m$), then

$$\begin{aligned} \hat{m}= & {} m\Big (\frac{\rho ^v_1}{\langle \rho ^v \rangle }\frac{l_1}{l_1 + l_2} + \frac{\rho ^v_2}{\langle \rho ^v \rangle }\frac{l_2}{l_1 + l_2}\Big ) = m \frac{\langle \rho ^v \rangle }{\langle \rho ^v \rangle } = m,\\ \Big (\frac{1}{H_1} + \frac{1}{H_2}\Big )= & {} h^{-1}\Big (\frac{\rho ^v_1}{\langle \rho ^v \rangle }\frac{l_1}{l_1 + l_2} + \frac{\rho ^v_2}{\langle \rho ^v \rangle }\frac{l_2}{l_1 + l_2}\Big ) = h^{-1}\frac{\langle \rho ^v \rangle }{\langle \rho ^v \rangle } = h^{-1}, \end{aligned}$$

and the dependency of expressions (22) and (23) on $\rho ^v_{1,2}$ vanishes.

In the special case of $\eta =1$, the conditions for PTW stability also simplify. The reduction to normal form of Eq. (11) (with $\delta = 1$) to Eq. (12) follows the script fully described by Sherratt et al. (2003) based on Guckenheimer and Holmes (2013). The first step in this script is to convert the linear part of the equations to normal form to give

24 $$\begin{aligned} \begin{aligned} \partial _T H&= \partial ^2_X H + \epsilon \Lambda _0 H + (\Omega _0 + \epsilon \Omega _1)P + \mathcal {G}(H,P), \\ \partial _T P&= \partial ^2_X P + \epsilon \Lambda _0 P - (\Omega _0 + \epsilon \Omega _1)H + \mathcal {F}(H,P), \end{aligned} \end{aligned}$$

with *H* and *P* being linearly transformed versions of *h* and *p*. $\mathcal {F}(H,P)$ and $\mathcal {G}(H,P)$ determine the stability of the PTW solutions (13) and are obtained with the aid of *Mathematica* [see section 2 of Andrade and Cobbold (2022)]. The variable $\epsilon = C-C_{\textrm{crit}}$ is assumed to be small (small-amplitude approximation). The expressions for $\Lambda _0, \Omega _0, \Omega _1$ are computed from the eigenvalues of the Jacobian matrix associated with Eq. (11) evaluated at the coexistence steady state.

The derivatives of $\mathcal {F}(H,P)$ and $\mathcal {G}(H,P)$ with respect to *H* and *P* are evaluated to calculate the expression of $\omega _1$ which determines the stability of the PTWs (see section 4 of Andrade and Cobbold (2022)). The full expression for $\omega _1$ is complicated and not shown, but in the special case of $\eta =1$, the expressions for $\mathcal {F}(H,P)$ and $\mathcal {G}(H,P)$ simplify significantly:

$$\begin{aligned} \mathcal {F}(H,P)= & {} H \left( \sqrt{\frac{E_1(1+\beta )-1}{B_1 E_1 (E_1(1+\beta )+1)}}\right. \\{} & {} \left. -\, \frac{((1+\beta ) E_1-1)^2 \left( \frac{(\beta +1) E_1}{(E_1(1+\beta )+1)^2}-P \sqrt{\frac{(1+\beta ) E_1-1}{B_1 E_1 (E_1(1+\beta )+1)}}\right) }{\sqrt{B_1 E_1} \left( \frac{(1+\beta ) E_1-1}{E_1(1+\beta )+1}\right) ^{3/2} ((\beta +1) E_1 (H+1)+H)}\right) ,\\ \mathcal {G}(H,P)= & {} -\frac{H \left( -P \left( (\beta +1)^2 E_1^2-1\right) \sqrt{\frac{E_1(1+\beta )-1}{B_1 E_1 ((1+\beta ) E_1+1)}}+H^2 (E_1(1+\beta )+1)+H\right) }{(\beta +1) E_1 (H+1)+H}. \end{aligned}$$

As these expressions only depend on predator residence index only through (22) and (23), we conclude that $\mathcal {F}, \mathcal {G}$ and consequently $\omega _1$ also do not depend on the predator residence index when $\eta = 1$, $m_1 = m_2$ and $h_1 = h_2$.

## Appendix C: Amplitude of the Spatially Uniform Predator Oscillations

See Fig. 11.

## Appendix D: Extra Numerical Integrations of the Homogenised System

In this section, we exhibit numerical integrations for other points of the plot from Fig. 5, complementing the plots in Fig. 6. Figure 12 shows numerical simulations of the homogenised equations (11) with parameters values estimated for the hare-lynx system, predicted to be stable for $\eta = 1$ and unstable for $\eta = 7$. These plots illustrate how longer domains are necessary to determine the stability of the PTWs, as considering short domains can make unstable solutions to be misclassified as stable (compare the solution of Fig. 12 for $\eta = 7, \epsilon = 0.5$ for $X \in [0,800]$ to the same solution for $X \in [0,7000]$), both for small and large amplitudes. However, the simulations of longer domains require very long computation times in order to transient effects to vanish. Similar challenges arise when simulating the equations for the parameter values of the weasel-vole system (Fig. 13), predicted to be unstable for $\eta = 1$ and $\eta = 7$.

Moreover, the ($E_1,B_1$) pairs shown in Figs. 12 and 13 are close to the stability boundaries of $\eta = 1$ and $\eta = 7$ in Fig. 5. This causes the solutions to exhibit long transients, which require even longer simulations. In contrast, in Fig. 14 we simulate Eq. (11) for a point in the ($E_1,B_1$)-space further away from the stability boundaries. By comparing left and right plots in Fig. 14, there is clear shift in stability even for a shorter domain, matching the prediction from Fig. 5 both for small and large amplitudes: unstable for $\eta = 1$ and stable for $\eta =7$.

## Appendix E: Implications of the $\delta =1$ Constraint

In this section, we prove that the $\delta =1$ constraint can be satisfied when the predators on each patch diffuse further than prey despite the homogenised landscape scale diffusion rates for prey and predator being equal.

From the expressions for $\hat{D}^{u,v}$ (Eq. 8), the $\delta =1$ condition yields

$$\begin{aligned} \hat{D^{u}}= & {} \hat{D^{v}} \implies \Big (\frac{\rho ^u_1 l_1 + \rho ^u_2 l_2}{l_1 + l_2}\Big )\Big (\frac{(1-\alpha ^u) l_1 + \alpha ^u l_2}{l_1 + l_2}\Big ) \\ {}= & {} \Big (\frac{\rho ^v_1 l_1 + \rho ^v_2 l_2}{l_1 + l_2}\Big )\Big (\frac{(1-\alpha ^v) l_1 + \alpha ^v l_2}{l_1 + l_2}\Big ). \end{aligned}$$

For simplicity, we assume that $l_1 = l_2$, with an analogous argument holding for general relative patch sizes. Under such an assumption, the expression becomes

$$\begin{aligned} \rho ^u_1 + \rho ^u_2 = \rho ^v_1 + \rho ^v_2 \implies \frac{1}{D^u_1 (1-\alpha ^u)} + \frac{1}{D^u_2 \alpha ^u} = \frac{1}{D^v_1 (1-\alpha ^v)} + \frac{1}{D^v_2 \alpha ^v}. \end{aligned}$$

We further assume that prey and predator have preference for different patch types such that $\alpha ^u = \alpha$ and $\alpha ^v = 1 - \alpha$. However, a similar argument still holds if $\alpha ^u > 0.5$ and $\alpha ^v < 0.5$, or if $\alpha ^u < 0.5$ and $\alpha ^v > 0.5$. The expression then becomes

25 $$\begin{aligned}{} & {} \frac{D^u_2\alpha + D^u_1(1-\alpha )}{D^u_2 D^u_1\alpha (1-\alpha )} = \frac{D^v_2(1-\alpha ) + D^v_1\alpha }{D^v_2 D^v_1\alpha (1-\alpha )} \implies \nonumber \\{} & {} \alpha (D_2^v - D_1^u)D_1^vD_2^u + (1-\alpha )(D_1^v- D_2^u)D_2^vD_1^u = 0. \end{aligned}$$

Since $0<\alpha <1$, expression (25) is satisfied if

(I): $D_2^v > D_1^u$ and $D_1^v < D_2^u$, or if
(II): $D_2^v < D_1^u$ and $D_1^v > D_2^u$, or if
(III): $$D_2^v = D_1^u$$ and $$D_1^v = D_2^u$$.

Case (I) is valid, e.g. if $D_1^u< D_1^v< D_2^u < D_2^v$ and case (II) if $D_2^u< D_2^v< D_1^u < D_1^v$. In either case, we have shown that it is possible to have $D_2^v > D_2^u$ and $D_1^v > D_1^u$ despite $\delta = 1$, provided predator and prey prefer different patch types.
